# Optimising ambulance service contribution to clinical trials: a phenomenological exploration using focus groups

**DOI:** 10.29045/14784726.2019.12.4.3.8

**Published:** 2019-12-01

**Authors:** Helen Pocock, Michelle Thomson, Sarah Taylor, Charles D. Deakin, Ed England

**Affiliations:** South Central Ambulance Service NHS Foundation Trust: ORCID iD: http://orcid.org/0000-0001-7648-5313; South Central Ambulance Service NHS Foundation Trust; South Central Ambulance Service NHS Foundation Trust; South Central Ambulance Service NHS Foundation Trust: ORCID ID: https://orcid.org/0000-0002-2565-9771; South Central Ambulance Service NHS Foundation Trust: ORCID iD: https://orcid.org/0000-0002-8009-2843

**Keywords:** cardiac arrest, ethics, out-of-hospital research, paramedic, pre-hospital trials

## Abstract

**Introduction:**

Out-of-hospital cardiac arrest trials can prove challenging and there is a need to share learning from those that have recruited successfully. We have just completed three years of recruitment to PARAMEDIC2, a placebo-controlled trial of adrenaline in out-of-hospital cardiac arrest. This study was designed to describe the experience of operational ambulance staff involved in recruiting patients into PARAMEDIC2.

**Methods:**

Four focus groups involving trial paramedics and supporting members of the emergency care team were conducted across different geographical regions of a single UK ambulance service participating in the PARAMEDIC2 study. Data analysis was supported by NVivo 12 and themes were identified using a thematic analysis approach.

**Results:**

Forty-four participants contributed to the focus groups. Four overarching themes were identified: *context for the research*, *ethical concerns*, *concerns at the patient’s side* and *ongoing trial support*. Participants felt that research such as PARAMEDIC2 is important and necessary to drive medical progress. They valued the opportunity to be part of a large project. Due to the deferred consent model employed, public awareness of the trial was felt to be important. Most expressed equipoise regarding adrenaline, but some felt concerned about enrolling younger patients and there was discussion around what constitutes a successful outcome. Struggles with ethical concerns were overcome through training and one-to-one discussion with research paramedics. Participants valued feedback on their performance of trial tasks, but also wanted feedback on their resuscitation skills. Cardiac arrest places a high cognitive demand on paramedics; simplicity and reinforcement of trial processes were key to facilitating recruitment. Caring for relatives was a high priority for paramedics and some felt conflicted about not discussing the trial with them.

**Conclusions:**

This study has provided insights into paramedic experience of a large-scale pre-hospital trial. Investment in time and resource to provide face-to-face training and personalised feedback to paramedics can foster engagement and optimise performance.

## Introduction

### Background

Over the past decade, UK ambulance services have begun to make a substantial contribution to the ever-expanding pre-hospital evidence base. There has been a positive change in culture and attitude towards research engagement within ambulance trusts, resulting in the recruitment of significant numbers of critically ill patients to large randomised studies, such as PARAMEDIC (Perkins et al., 2014), AIRWAYS-2 (Benger et al., 2018) and RIGHT-2 (RIGHT-2 Investigators, 2019). For three years, the South Central Ambulance Service was engaged in the pre-hospital assessment of the role of adrenaline: measuring the effectiveness of drug administration in cardiac arrest (PARAMEDIC2) trial, the largest out-of-hospital cardiac arrest medicines trial in Europe to date (Perkins et al., 2018).

Despite its inclusion in cardiac arrest treatment protocols for over 50 years, there is a lack of reliable data supporting the use of adrenaline. While it may improve the chance of restoring circulation in the short term, evidence was lacking for an improved chance of survival to hospital discharge or good neurological outcomes (Morrison et al., 2010). In this trial, paramedics delivered either standard adrenaline or a placebo (sodium chloride 0.9%) to patients in cardiac arrest. All other aspects of the resuscitation protocols were followed as normal, including recognition of death where appropriate. Individual randomisation was achieved by opening a pre-randomised treatment pack at the patient’s side. A waiver of consent was granted for the point of treatment; consent for follow-up was sought from survivors in hospital (Perkins et al., 2016).

The success of these studies is largely determined by paramedic engagement and the subsequent willingness to recruit patients under their care. Ethical concerns, such as the need for informed consent, and possible delays to patient treatment associated with research procedures, have previously led paramedics to feel conflicted about research involvement (Burges Watson et al., 2012). Patient recruitment in PARAMEDIC2 was successful, in contrast to other similar studies (e.g. Jacobs, Finn, Jelinek, Oxer, & Thompson, 2011). It is therefore important to identify factors that were key to successful implementation and identify processes that could be further improved.

Most previous research has identified what helped and hindered research from the perspective of the research team (McClelland, Pennington, Byers, Russell, & Lecky, 2015; Shaw et al., 2014). The views of paramedics who recruited patients have previously been captured by quantitative questionnaire (Pocock et al., 2016). The aim of this study was to describe the experience of paramedics and supporting members of the emergency care team involved in recruiting patients in cardiac arrest to a medicines trial, focusing on barriers and facilitators to successful patient recruitment.

## Methods

### Study design

Individual paramedics’ experiences of recruiting patients, as well as ambulance staff culture, were explored using focus groups. This allowed the researcher to observe group members’ reactions to what was said. This type of discussion group can help people clarify their views and encourage participation of nervous speakers as they are among friends (Kitzinger, 1995).

### Philosophical underpinnings

This study sits within the interpretive paradigm, and a hermeneutic phenomenological approach was adopted to understand participants’ interpretations of the lived experience of being involved in a trial. This approach utilises the particular knowledge and experience of the researchers in both detecting and interpreting meaning in the data. Researchers had previously managed the PARAMEDIC2 trial and so were very familiar with the culture and practices of the study population. To avoid any indirect influence or acquiescence, focus groups were facilitated by a research paramedic based in a different geographic area from that of the participants.

### Setting

The study was conducted in a regional NHS ambulance service in the south of England serving a population of 4 million. All front line emergency ambulance staff attended trial training to foster awareness of the trial, since resuscitation requires teamwork. A total of 759 paramedics recruited 2488 patients, which contributed approximately 30% of the total national recruitment.

### Participants

We sought the views of front line ambulance staff trained to recruit, or support recruitment of, patients to the PARAMEDIC2 trial. Staff who did not recruit patients were included as they may have been an integral part of the team treating the patient. Each focus group was a naturally occurring team of ambulance staff (working similar shifts and training together). Teams where at least 50% of paramedic members recruited a patient and held a training session within the study period (June–July 2018) were invited to take part. Purposive sampling was used to select teams from diverse locations throughout the Trust.

### Study size

Using naturally occurring groups which comprised 12–15 members, but allowing for absence/choice not to take part, it was anticipated that approximately 8–10 participants would attend each session, which is the ideal recommended group size (Kitzinger, 1995). Sessions lasted approximately one hour. Previous ambulance service focus group research identified no new topics after the third focus group (Shaw & Siriwardena, 2014). In case we did not reach saturation at this point, we planned four sessions.

### Data collection

A topic guide was used to start discussion (Supplementary 1) and to re-start it if the conversation ‘dried up’. This was tested and adapted using a pilot focus group. Focus groups were facilitated by research paramedics involved with delivering the trial. This strategy has been used successfully previously (Johnson et al., 2017). Sessions were audio recorded and transcribed by a research administrator and a research paramedic. Transcriptions were anonymised, then checked by a different research paramedic.

### Data analysis

Data were analysed according to the six phases of thematic analysis (Braun & Clarke, 2006). (1) Data familiarisation was achieved by listening to audio files and checking and reading through the transcriptions. (2) Multiple coding sweeps were undertaken (by HP), firstly to code for surface and then for latent meaning. (3) Themes were generated (by HP) and then (4) reviewed (by EE and ST) and (5) named, before (6) a final report was produced. Data analysis was supported by NVivo 12.

Findings were presented to, and checked by, the focus groups to ensure validity.

## Results

Four focus groups were conducted, comprising nine to twelve members, with a total of 44 participants. One hundred per cent of paramedics in three teams and 75% in one team had recruited patients into the trial. Teams included paramedics, specialist paramedics, ambulance technicians and emergency care assistants. Only paramedics and specialist paramedics could recruit patients to the PARAMEDIC2 trial, as the other staff groups do not have professional registration, but all grades were involved in the resuscitation of patients enrolled in the trial.

Forty-four codes were initially identified. Codes that were conceptually similar were merged, for example ‘prolonging harmful resuscitation’ and ‘avoiding a condition worse than death’. No new codes were identified by the fourth focus group so it was assumed that data saturation had been reached. Initially nine themes were identified. Following discussion within the research team and re-visiting the data, this number was revised to 12 and grouped into themes and sub-themes as shown below.

Four overarching themes were identified in the focus group sessions: context for the research, ethical concerns, concerns at the patient’s side and ongoing trial support (Box 1).

**Box 1. Themes and subthemes identified.**

Themes and subthemes

1. *Context for the research*
  The role of research
  Inclusion
2. *Ethical concerns*
  Equipoise
  Public awareness and prior expression of wishes
  Patient outcomes
  Resolved ethical concern
3. *Concerns at the patient’s side*
  Managing cognitive demands
  Feelings about recruitment
  Openness with families
4. *Ongoing trial support*
  Simplicity
  Reinforcement of training
  Desire for validation

### Context for the research

#### The role of research

Within this theme, staff talked about the role of research in general and its necessity to drive medical progress. The majority were keen to test the effectiveness of the treatments they deliver in routine practice and to make changes to practice if indicated. They felt it was important to be involved in PARAMEDIC2, with some identifying that they had anticipated such a trial.

We’ve been administering a cardiac drug as … a matter of course without knowing whether it is the right thing to do or the wrong thing to do and [the] only way to establish something like that is by a, sort of … trial such as this. **[FG2, P5]**

Although participants were enthusiastic about research, they acknowledged that it was something new; paramedic practice has traditionally been heavily protocol-driven, with little awareness of how protocols are developed. This change provoked anxiety in a small number of participants.

It was scary because it was working against our, what we have always done, what we were used to doing I suppose. Um … It’s a new concept … and change is always difficult. **[FG2, P1]**

#### Inclusion

A majority of participants agreed that they felt included, supported and part of something bigger. One participant had previously worked in a neighbouring trust, also engaged in the trial, and liked the fact this was a point of commonality.

Everybody was included; it wasn’t just one area. I felt actually that’s quite supportive and positive. **[FG3, P1]**

### Ethical concerns

#### Equipoise

While most paramedics expressed equipoise about adrenaline, it was evident that this was not universally the case, with one referring to adrenaline as ‘the right drug’ and another equating trial medicines with placebo.

… you don’t know what drug you were using, be it the adrenaline or placebo, and [when] you get no response from that patient, um I suppose it is natural to wonder, if … it was adrenaline, would that have changed something? **[FG4, P1]**

Many of the ethical concerns were linked to participants’ assumptions about the (short-term) actions of adrenaline and they therefore felt it was wrong to withhold this treatment. These concerns were heightened when treating younger adults:

The younger patient … there is more personal … sort of, you get a bit more emotionally involved and I think we would all want to think that we were doing the right thing. Umm, but then I suppose we don’t know what the right thing is which is why we have got this trial. **[FG4, P3]**

#### Public awareness and prior expression of wishes

Overall, participants felt it was important that members of the public were made aware of the trial. Some participants felt that the public had been well informed, with a number reporting they had seen television news coverage of the trial. A minority were concerned there should have been more publicity or that publicity may not have been noticed.

I saw something in my local chemist, but it was just a poster on the bottom of the counter, so I don’t know how well, if people didn’t want to be part of the trial … how well they would have known about it. **[FG3, P1]**

#### Patient outcomes

There was much discussion about what society would or should value as an outcome. Some felt life should be preserved at all costs, whereas others expressed a view that sometimes life-saving treatments can be inappropriate.

But that’s the sort of stuff that they looked at when they went to the ethics committee, isn’t it? That’s the risk they are taking to find out whether or not we are actually just ridiculously, um, putting people in ICU and nursing homes with zero quality of life when in reality they would be better off dead. [P6]

But then that’s you playing God, but it comes back to the morals, who’s to say? [P3]

But as a country, we’re bigger. We’re into dignified deaths. [P1] **[FG1, P6, P3, P1]**

#### Resolved ethical concern

Some participants, who originally struggled with ethical concerns, described how they overcame these worries through attending training sessions or talking face-to-face with research paramedics. One paramedic described how their views changed from feeling that the trial was unethical to, later, feeling that it was unethical to not take part as this would deny patients the chance of recruitment.

From the, um, initial briefing, so, when [the research paramedic] was talking to us about the trial … I found that quite informative and, er, put a lot of the problems that I had in my brain about it … to rest because we were able to have quite a good open discussion about why we were doing the trial. **[FG3, P3]**

### Concerns at the patient’s side

#### Managing cognitive demands

Most paramedics have a low exposure to cardiac arrests, and such incidents place a high cognitive demand on all team members (Perkins et al., 2014). Participants talked also about emotional demands, which are heightened when dealing with younger adults in cardiac arrest.

… it’s a stressful umm, anxious, emotive situation to be involved in. **[FG4, P1]**

… especially in those, in that high-pressure situations, you know you got family, you got everything round you and the last thing you want to be doing is, you know … an extra process. **[FG1, P3]**

#### Feelings about recruiting patients

Participants commonly described their feelings about being involved in the trial. While most were enthusiastic about their involvement, a few expressed anxiety with regard to specific scenarios. Although younger patients were a particular concern for staff, this was not necessarily linked to the trial, but to the condition of interest.

… it is more stressful in a younger patient than the patient at the end of their sort of, you know, ‘normal life’. **[FG4, P3]**

#### Openness with families

One important role in managing a cardiac arrest is communicating with families. Due to the waiver of consent model employed, a minority of participants reported having to manage internal conflict, at times feeling that they were deceiving relatives.

There’s a small part of me that felt quite guilty when they said ‘oh please do whatever you can’ … and you say ‘Of course we will’ but there’s a tiny part of me that knows full well you won’t be, you’re deliberately lying to the patients because you know that you’re going to be giving them a research trial pack. **[FG4, P1]**

This was challenged by the majority, recognising the necessity of the waiver of consent at that difficult and emotionally charged time.

… it is not the place or time to be having that sort of discussion because you don’t have the time to get informed consent … to go through the arguments. **[FG4, P2]**

### Ongoing trial support

#### Simplicity

Participants recognised that support was necessary and described different ways it was provided as well as highlighting ways it could be improved. Staff talked about the importance of making training and procedures simple, since the high cognitive load when managing cardiac arrest gave them little capacity for additional tasks. Simplicity, as a theme, ran through all aspects of the trial process.

… it all seemed a little bit confusing, different labels being stuck on different bags, but if you did this then that label had to go on that one and, you know, we’re just ambulance folk … who just like our … simple ways, especially in those, in those high-pressure situations. **[FG1, P3]**

#### Reinforcement of training

Paramedics required reinforcement of trial process and procedures due to the time lag between training and patient recruitment, which in some cases could be substantial. It helped if processes were as close to normal practice as possible.

Reinforcement and reminders were provided in a variety of different ways, such as on pocket cards carried by the crews, stickers on medicine packs and stickers in the vehicle cabs, all of which were available at the point of need.

So it meant that whilst you were on the way to a job, you [could] look at it and go ‘right, ok’. It’s … fresh in your head … the exclusion … and the inclusion criteria, so it was good, it wasn’t like you were having to sort of go ‘Oh, where’ve I put it? Oo, which email was it?’ because it was there in front of you. **[FG3, P1]**

This was equally important for some of the post-recruitment processes, which staff found difficult to remember.

… you use all the drugs and do the arrest, and then you go, ‘Oh what do I do now?’ … I couldn’t remember any of it without [the pocket card]. **[FG2, P2]**

#### Desire for validation

The importance of receiving feedback was universally recognised, to confirm that procedures had been correctly followed. Participants also highlighted the importance of immediate feedback after reporting a recruitment, to acknowledge receipt.

What was quite nice after you’d done one, to get that little letter at the end just saying thank you for enrolling a patient … if it’s right or if you had forgotten little details, like [for] next time? **[FG3, P5]**

Crews also wanted feedback on their performance of the basics at a cardiac arrest.

I think the little gadget to sort of check your compressions, umm … perhaps with hindsight might have been needed at the beginning, because part of the trial is checking how good your basic life support is. **[FG2, P8]**

## Discussion

Despite initial concerns that the ethically contentious nature of the trial might make paramedics wary of taking part, this was not the case. PARAMEDIC2 demonstrated that it was possible for a geographically dispersed, mobile workforce to recruit large numbers of patients over a long recruitment period, factors previously thought of as challenges (McClelland et al., 2015). The scale of the research was perceived positively since it fostered feelings of inclusion and being valued. These feelings have previously been linked with individuals’ positive feelings about, and willingness to invest in, a team (Ellemers, Sleebos, Stam, & de Gilder, 2013). Viewing a trial as a team endeavour, widening participation to include as many paramedics/stations/regions as possible and fostering connections with others involved may maximise the engagement of individual paramedics.

Emergency research presents unique ethical challenges and requires the building and maintenance of trust between the research community and the public (Nelson et al., 2013). A lack of honesty in the patient–paramedic relationship as a consequence of being blinded to treatment allocation has previously been identified as a concern of UK paramedics conducting blinded trials and a threat to professional identity (Burges Watson et al., 2012). In the focus groups, staff indicated that it was important to promote awareness of the trial. Having information in the public domain helped staff to overcome the feeling of withholding information from relatives. The largely well-received public awareness campaign provided reassurance to many of a general acceptance of the trial and maintenance of public trust.

Staff also felt it important that people should have the opportunity to express their wishes prior to recruitment since they would lack capacity at the time of recruitment. During the trial, ambulance staff had been trained to look for ‘NO STUDY’ bracelets, which members of the public could request in response to trial publicity. A range of public awareness campaigns was felt to be important to those recruiting to this waiver of consent trial.

Participants benefitted from face-to-face training and conversations with research paramedics in overcoming their ethical concerns. This helped to clarify their understanding of the risks and benefits associated with the treatment under investigation. The description by one participant of a moral imperative to take part so that patients are not denied the opportunity to be included is consistent with current UK policy, which advocates equality of opportunity for involvement in research for all patients (Health Research Authority, 2017).

Most paramedics in the focus groups articulated equipoise with respect to adrenaline. A small number indicated a possible lack of equipoise (evident from referral to adrenaline as ‘the right drug’). Although *clinical* equipoise is the state of uncertainty assumed in the design of a randomised controlled trial, *personal* equipoise only exists if the recruiting clinician genuinely has no pre-conceived preference for the treatment options (Cook & Sheets, 2011). In critical care research, lack of individual equipoise can result in ‘surreptitious opposition’ which may lead to problems in recruitment (Pattison, Arulkumaran, Humphreys, & Walsh, 2017). Paramedics are used to seeing short-term outcomes (e.g. survival) and rarely find out about their patients’ progress ‘beyond the resuscitation room’. It may therefore be harder for this professional group to have personal equipoise regarding long-term outcome (e.g. the primary outcome of 30 days in PARAMEDIC2), which is an important consideration for those designing training packages for pre-hospital research.

In common with previous research, in certain cases there were differences of opinion regarding the appropriateness of resuscitation (Anderson, Gott, & Slark, 2018). This reflects a wider societal debate but is not in conflict with the aim of the trial, that of optimising resuscitation. Although there was some discussion regarding what constitutes a good resuscitation outcome, there was no suggestion that paramedics’ own beliefs influenced their involvement in the trial. Personally held beliefs have previously been found to have less influence on decisions regarding resuscitation than professional experience, training and the legal framework (Leibold, Lassen, Lindenberg, Graf, & Wiese, 2018).

Managing cardiac arrest places high cognitive demands on paramedics. Eye tracking technology is starting to reveal the broad range and complexity of cognitive tasks undertaken when managing a cardiac arrest situation in a hospital emergency department setting (White et al., 2018). In the out-of-hospital setting with its uncontrolled environment and restricted number of personnel, the cognitive load is likely to be even higher, leaving little capacity for additional tasks such as research delivery. Paramedics are also subject to emotional demands during such situations (Anderson et al., 2018). They are often faced with the emotional response of family or bystanders (Waldrop, Clemency, Lindstrom, & Clemency, 2015). Several participants felt that they were not being entirely honest with relatives on scene. Other group members observed that, due to their already distressed state, discussion of the trial at this time would be inappropriate. Caring for relatives has previously been defined by paramedics as the most important aspect of care delivery during a resuscitation (Steen, Naess, & Steen, 1997). Dealing with this demanding aspect of care will continue to be a dilemma for future trials and will need thorough consideration in the context of the relevant legal and ethical frameworks.

Participants appreciated the simplicity of the trial procedures, as in previous studies (Ankolekar, Parry, Sprigg, Siriwardena, & Bath, 2014; Pocock et al., 2016). This made it easier to integrate into a complex clinical context. Critical care research has been hampered by complexity, with teams opting not to take part in trials considered too complex. It is important, therefore, to keep processes simple in pre-hospital research.

‘Refresher’ training, provided annually to all operational teams, helped to remind staff of trial procedures and reinforced feelings of simplicity. We identified the importance of regular communication with staff, in keeping with previous UK pre-hospital studies (Ankolekar et al., 2014; McClelland et al., 2015). Paramedics welcomed feedback on how well they followed trial procedures and requested feedback on their delivery of basic life support. This is in contrast with previous research that identified concerns and prejudice regarding the employment of real-time CPR feedback devices (Brinkrolf et al., 2018). Rather than feeling threatened, participants in this study appreciated the personalised feedback and recognised the importance of optimising all aspects of care, not only those under investigation.

This study suggests that the PARAMEDIC2 trial offered advantages to paramedics that encouraged their participation. Firstly, proactive publicity following public consultation reassured paramedics that the trial was within the public domain and, although perhaps not uncontentious, was not unacceptable to the public. Secondly, the scale of the research, aided by the fact that UK emergency ambulance services are part of the NHS, was a reassurance to paramedics.

### Limitations

Natural groups were used in the focus groups. This was largely for convenience but also because members of these groups had undertaken the activity of interest together. Although this should have put participants at ease, existing hierarchies and relationships might have prevented some members from speaking up.

Despite attempts to minimise the effect of any existing power imbalance/relationship by ensuring that researchers were from a different area from that of the team and did not wear uniform, this may not have been fully achieved. Negative views and opinions *were* expressed but it is not known whether those who made a lesser contribution felt inhibited.

## Conclusions

This study has provided insights into paramedic experience of being involved in a large-scale pre-hospital trial. Facilitators included fostering feelings of inclusion and being valued which was achieved through widening participation in the trial to include the whole Trust. Personalised feedback to paramedics furthered their engagement and maintained enthusiasm. The achievement of personal equipoise was a facilitator to patient recruitment and highlights the importance of education around the background and need for the trial.

Barriers included an evident lack of personal equipoise in some paramedics, leading them to question the ethics of the trial. This was minimised through investment in time and resource to provide face-to-face training which helped to address ethical concerns. The cognitive demands of recruiting patients were minimised through simplification of trial procedures and the reinforcement of training. The emotional demands of providing care to patients and relatives weighed heavily on some paramedics. Activities that helped to preserve public trust such as development of public awareness of the trial were felt to be important by paramedics.

The findings of this study could help triallists optimise the performance of future pre-hospital trials.

## Author contributions

All authors conceptualised the paper and designed the service evaluation and analyses. HP, MT, ST and EE performed the analyses. HP drafted the paper. All authors contributed to the interpretation of the results and revising the paper. EE is the guarantor of the work.

## Conflict of interest

None declared.

## Ethics

Health Research Authority approval was obtained (IRAS no. 244991) and informed consent was obtained from participants prior to commencement of each focus group.

## Funding

This study was funded by the College of Paramedics’ small grant scheme. The PARAMEDIC2 trial was funded by the National Institute for Health Research’s Health Technology Assessment programme (project number 12/127/126). The views and opinions expressed therein are those of the authors and do not necessarily reflect those of the Health Technology Assessment Programme, NIHR, NHS or the Department of Health.

## References

[bibr_1] Anderson N. E. Gott M. Slark J. (2018). Grey areas: New Zealand ambulance personnel’s experience of challenging resuscitation decision-making. *International Emergency Nursing*, 39, 62–67. 28882750 10.1016/j.ienj.2017.08.002

[bibr_2] Ankolekar S. Parry R. Sprigg N. Siriwardena A. N. Bath P. M. W. (2014). Views of paramedics on their role in an out-of-hospital ambulance-based trial in ultra-acute stroke: Qualitative data from the Rapid Intervention with Glyceryl trinitrate in Hypertensive stroke Trial (RIGHT). *Annals of Emergency Medicine*, 64, 640–648. 24746844 10.1016/j.annemergmed.2014.03.016

[bibr_3] Benger J. R. Kirby K. Black S. Brett S. J. Clout M. Lazaroo M. J. … Rogers C. A. (2018). Effect of a strategy of a supraglottic airway device vs tracheal intubation during out-of-hospital cardiac arrest on functional outcome: The AIRWAYS-2 randomized clinical trial. *Journal of the American Medical Association*, 320, 779–791. 30167701 10.1001/jama.2018.11597 PMC6142999

[bibr_4] Braun V. Clarke V. (2006). Using thematic analysis in psychology. *Qualitative Research in Psychology*, 3, 77–101.

[bibr_5] Brinkrolf P. Lukas R. Harding U. Thies S. Gerss J. Van Aken H. … Bohn A. (2018). A better understanding of ambulance personnel’s attitude towards real-time resuscitation feedback. *International Journal for Quality in Health Care*, 30, 110–117. 29340631 10.1093/intqhc/mzx189

[bibr_6] Burges Watson D. L. Sanoff R. Mackintosh J. E. Saver J. L. Ford G. A. Price C. … Murtagh M. J. (2012). Evidence from the scene: Paramedic perspectives on involvement in out-of-hospital research. *Annals of Emergency Medicine*, 60, 641–650. 22387089 10.1016/j.annemergmed.2011.12.002 PMC3690919

[bibr_7] Cook C. Sheets C. (2011). Clinical equipoise and personal equipoise: Two necessary ingredients for reducing bias in manual therapy trials. *Journal of Manual and Manipulative Therapy*, 19, 55–57. 22294855 10.1179/106698111X12899036752014 PMC3172958

[bibr_8] Ellemers N. Sleebos E. Stam D. de Gilder D. (2013). Feeling included and valued: How perceived respect affects positive team identity and willingness to invest in the team. *British Journal of Management*, 24, 21–37.

[bibr_9] Health Research Authority. (2017). *UK policy framework for health and social care research*. Retrieved from http://www.ct-toolkit.ac.uk/news/uk-policy-framework-health-and-social-care/7251.

[bibr_10] Jacobs I. G. Finn J. C. Jelinek G. A. Oxer H. F. Thompson P. L. (2011). Effect of adrenaline on survival in out-of-hospital cardiac arrest: A randomised double-blind placebo-controlled trial. *Resuscitation*, 82, 1138–1143. 21745533 10.1016/j.resuscitation.2011.06.029

[bibr_11] Johnson M. O’Hara R. Hirst E. Weyman A. Turner J. Mason S. … Siriwardena A. N. (2017). Multiple triangulation and collaborative research using qualitative methods to explore decision making in pre-hospital emergency care. *BMC Medical Research Methodology*, 17, 11. doi: 10.1186/s12874-017-0290-z. 28118817 10.1186/s12874-017-0290-z PMC5259953

[bibr_12] Kitzinger J. (1995). Qualitative research: Introducing focus groups. *BMJ*, 311, 299. doi: https://doi.org/10.1136/bmj.311.7000.299. 7633241 10.1136/bmj.311.7000.299 PMC2550365

[bibr_13] Leibold A. Lassen C. L. Lindenberg N. Graf B. M. Wiese C. H. (2018). Is every life worth saving: Does religion and religious beliefs influence paramedics’ end-of-life decision-making? A prospective questionnaire-based investigation. *Indian Journal of Palliative Care*, 24, 9–15. 29440799 10.4103/IJPC.IJPC_128_17 PMC5801638

[bibr_14] McClelland G. Pennington E. Byers S. Russell W. Lecky F. (2015). The challenges of conducting prehospital research: Successes and lessons learnt from the Head Injury Transportation Straight to Neurosurgery (HITS-NS) trial. *Emergency Medicine Journal*, 32, 663–664. 25573067 10.1136/emermed-2014-203870

[bibr_15] Morrison L. J. Deakin C. D. Morley P. T. Callaway C. W. Kerber R. E. Kronick S. L. … Advanced Life Support Chapter Collaborators. (2010). Part 8: Advanced life support: 2010 international consensus on cardiopulmonary resuscitation and emergency cardiovascular care science with treatment recommendations. *Resuscitation*, 81, e93–e174. 20956032 10.1016/j.resuscitation.2010.08.027

[bibr_16] Nelson M. J. Dlorio N. M. Schmidt T. A. Zive D. M. Griffiths D. Newgard C. D. (2013). Why persons choose to opt out of an exception from informed consent cardiac arrest trial. *Resuscitation*, 84, 825–830. 23402968 10.1016/j.resuscitation.2013.01.030 PMC3654061

[bibr_17] Pattison N. Arulkumaran N. Humphreys S. Walsh T. (2017). Exploring obstacles to critical care trials in the UK: A qualitative investigation. *Journal of the Intensive Care Society*, 18, 36–46. 28979535 10.1177/1751143716663749 PMC5606357

[bibr_18] Perkins G. D. Ji C. Deakin C. D. Quinn T. Nolan J. P. Scomparin C. … PARAMEDIC2 Collaborators. (2018). A randomised controlled trial of epinephrine in out-of-hospital cardiac arrest. *New England Journal of Medicine*, 379, 711–721. 30021076 10.1056/NEJMoa1806842

[bibr_19] Perkins G. D. Lall R. Quinn T. Deakin C. D. Cooke M. W. Horton J. … Gates S. (2014). Mechanical versus manual chest compression for out-of-hospital cardiac arrest (PARAMEDIC): A pragmatic, cluster randomised controlled trial. *Lancet*, 385, 947–955. 25467566 10.1016/S0140-6736(14)61886-9

[bibr_20] Perkins G. D. Quinn T. Deakin C. D. Nolan J. P. Lall R. Slowther A.-M., … Gates S. (2016). Pre-hospital assessment of the role of adrenaline: Measuring the effectiveness of drug administration in cardiac arrest (PARAMEDIC-2) trial protocol. *Resuscitation*, 108, 75–81. 27650864 10.1016/j.resuscitation.2016.08.029 PMC5081174

[bibr_21] Pocock H. Deakin C. D. Quinn T. Perkins G. D. Horton J. Gates S. (2016) Human factors in prehospital research: Lessons from the PARAMEDIC trial. *Emergency Medicine Journal*, 3, 562–568. 10.1136/emermed-2015-204916 26917497

[bibr_22] RIGHT-2 Investigators. (2019). Prehospital transdermal glyceryl trinitrate in patients with ultra-acute presumed stroke (RIGHT-2): An ambulance-based, randomised, sham-controlled, blinded, phase 3 trial. *Lancet*, 393, 1009–1020. 30738649 10.1016/S0140-6736(19)30194-1 PMC6497986

[bibr_23] Shaw D. Siriwardena A. N. (2014). Identifying barriers and facilitators to ambulance service assessment and treatment of acute asthma: A focus group study. *BMC Emergency Medicine*, 14, 18. doi: 10.1186/1471-227X-14-18. 25086749 10.1186/1471-227X-14-18 PMC4125344

[bibr_24] Shaw L. Price C. McLure S. Howel D. McColl E. Younger P. Ford G. A. (2014). Paramedic initiated Lisinopril for acute stroke treatment (PIL-FAST): Results from the pilot randomised controlled trial. *Emergency Medicine Journal*, 31, 994–999. 24078198 10.1136/emermed-2013-202536 PMC4251169

[bibr_25] Steen E. Naess A. C. Steen P. A. (1997). Paramedics organizational culture and their care for relatives of cardiac arrest victims. *Resuscitation*, 34, 57–63. 9051825 10.1016/s0300-9572(96)01045-3

[bibr_26] Waldrop D. P. Clemency B. Lindstrom H. A. Clemency C. (2015). ‘We are strangers walking into their life-changing event’: How prehospital providers manage emergency calls at the end of life. *Journal of Pain and Symptom Management*, 5, 328–334. 10.1016/j.jpainsymman.2015.03.001 25828561

[bibr_27] White M. R. Braund H. Howes D. Egan R. Gegenfurtner A. Van Merrienboer J. G. Szulewski A. (2018). Getting inside the expert’s head: An analysis of physician cognitive processes during trauma resuscitations. *Annals of Emergency Medicine*, 72, 289–298. 29699720 10.1016/j.annemergmed.2018.03.005

